# Cancers Attributable to Alcohol Consumption in Nigeria: 2012–2014

**DOI:** 10.3389/fonc.2017.00183

**Published:** 2017-08-24

**Authors:** Michael Kolawole Odutola, Elima E. Jedy-Agba, Eileen O. Dareng, Sally N. Adebamowo, Emmanuel A. Oga, Festus Igbinoba, Theresa Otu, Emmanuel Ezeome, Ramatu Hassan, Clement A. Adebamowo

**Affiliations:** ^1^Institute of Human Virology, Abuja, Nigeria; ^2^Department of Non-communicable Disease Epidemiology, London School of Hygiene and Tropical Medicine, London, United Kingdom; ^3^Center for Cancer Genetic Epidemiology, Department of Public Health and Primary Care, University of Cambridge, Cambridge, United Kingdom; ^4^Department of Epidemiology and Public Health, Marlene and Stewart Greenebaum Comprehensive Cancer Center, University of Maryland School of Medicine, Baltimore, MD, United States; ^5^Battelle Memorial Institute, Baltimore, MD, United States; ^6^National Hospital Abuja, Abuja, Nigeria; ^7^University of Abuja Teaching Hospital, Gwagwalada, Nigeria; ^8^University of Nigeria Teaching Hospital, Enugu, Nigeria; ^9^Federal Ministry of Health, Abuja, Nigeria; ^10^Institute of Human Virology, University of Maryland School of Medicine, Baltimore, MD, United States

**Keywords:** cancer, alcohol, associated cancers, attributable cancers, cancer registry, Nigeria

## Abstract

**Introduction:**

Alcohol consumption has been identified as a risk factor for many cancers but less attention has been paid to the fraction of those cancers that are attributable to alcohol consumption. In this study, we evaluated the incidence and population attributable fraction (PAF) of cancers associated with alcohol consumption in Nigeria.

**Methods:**

We obtained data on incidence of cancers from two population-based cancer registries (PBCRs) in Nigeria and identified cancer sites for which there is strong evidence of an association with alcohol consumption based on the International Agency for Research on Cancer Monograph 100E. We computed the PAF for each cancer site by age and sex, using prevalence and relative risk estimates from previous studies.

**Results:**

Between 2012 and 2014 study period, the PBCRs reported 4,336 cancer cases of which 1,627 occurred in males, and 2,709 occurred in females. Of these, a total of 1,808 cancer cases, 339 in males and 1,469 in females, were associated with alcohol intake. The age standardized incidence rate (ASR) of alcohol associated cancers was 77.3 per 100,000. Only 4.3% (186/4,336) of all cancer cases or 10.3% (186/1,808) of alcohol associated cancers were attributable to alcohol consumption. Some 42.5% (79/186) of these cancers occurred in males while 57.5% (107/186) occurred in females. The ASR of cancers attributable to alcohol in this population was 7.2 per 100,000. The commonest cancers attributable to alcohol consumption were cancers of the oral cavity and pharynx in men and cancer of the breast in women.

**Conclusion:**

Our study shows that 4.3% of incident cancers in Nigeria can be prevented by avoiding alcohol consumption. While the incidence of cancers associated with alcohol intake is high, the proportion attributable to alcohol consumption is much lower suggesting that the number of cancers that may be prevented by eliminating alcohol intake in this population is relatively low.

## Introduction

Alcohol consumption is a major risk factor for morbidity, disability, and mortality worldwide (1). In 2012, an estimated 3.3 million deaths (5.9% of all global deaths) were attributable to alcohol consumption, mainly from cardiovascular diseases, injuries, gastrointestinal illnesses, and cancer (2). According to the 2014 World Health Organization (WHO) Global Status Report on Alcohol and Health, 38.1% of the world’s population aged 15 years or older were estimated to be regular drinkers, with an average consumption of 38.6 g of pure alcohol per day (approximately 2.8 standard servings of alcoholic drinks) (2, 3).

There is considerable variation in the prevalence of drinking across the world, but the burden of disease and death remains significant in most countries (4). According to the WHO, in 2014, Nigeria was the fourth leading African country in alcohol consumption after South Africa, Namibia, and Gabon (2). The average national adult per capita consumption of alcohol between 2008 and 2010 in Nigeria was 10.1 l/year which is similar to that of the United States (9.8 l/year), but higher than that reported in China (6.7 l/year) (2).

The International Agency for Research on Cancer (IARC) and World Cancer Research Fund have concluded that there is strong evidence for a causal relationship between alcohol consumption and cancers of the oral cavity, pharynx, esophagus, colon, rectum, liver, gall bladder, pancreas, larynx, and breast (5, 6). According to IARC, 770,000 (5.5%) of the 14.1 million new cancer cases reported worldwide in 2012 were attributable to alcohol consumption (4, 7). In contrast, consumption of mild to moderate quantities of alcohol has an inverse association with risk of thyroid cancer (8).

The proportion of incident cancer cases and cancer deaths attributable to alcohol consumption varies across the world. In the African region, it is estimated that 4.8% of cancer cases were attributable to alcohol consumption in 2012, while in the American and European regions, 4.2 and 5.4% of cancer cases were attributable to alcohol consumption, respectively (4). Although country-specific estimates of cancers attributable to alcohol consumption are available for countries like Korea (2.0%), Australia (2.8%), United States (3.0%), United Kingdom (3.6%), and China (4.4%), there are no country-specific estimates of cancers attributable to alcohol consumption in Nigeria or other African countries (9–13).

With increasing economic development and disposable income, consumption of alcohol in many African countries is rising. It is therefore important to estimate the current burden of alcohol associated and alcohol attributable cancers in Nigeria in order to ascertain the impact of secular changes in alcohol consumption and the proportion of cancers that would be eliminated by reducing or prohibiting alcohol intake in this population. This would be helpful in guiding public health policies geared toward cancer prevention and control.

## Materials and Methods

### Data Sources

We obtained data on all incident cancer cases from 2012 to 2014 reported by two population-based cancer registries (PBCRs), the Abuja, and Enugu cancer registry (ECR) in Nigeria. The Abuja cancer registry (ABCR) is located in Abuja, the capital of Nigeria which is centrally located and home to people of varied ethnic groups and religions. ABCR was established in 2009 and its catchment area covers the entire federal capital territory which has a population of 1,406,239 people (14). The ECR is located in Enugu in the southeastern region of Nigeria. ECR was established in 2012, and covers a defined population of 1,103,153 people (14). The two PBCRs use the International Classification of Disease for Oncology, 3rd Edition (ICD-O3) for coding and classification of cancers. ABCR uses CanReg4 for data entry, processing, and storage, while ECR uses CanReg5 software. Data from the two registries analyzed in this study are based on de-identified data as is the international practice in cancer registration.

### Data Handling and Statistical Analysis

We performed data quality control checks to remove duplicates and to ensure logical correctness and overall accuracy of the data. Analysis of incident cancer cases and age standardized incidence rate (ASR) calculation was generated by the CanReg5 software. We identified cancer sites for which there is strong evidence of an association with alcohol consumption based on the IARC Monograph 100E (5). The cancer sites (ICD-O code) considered in this study were: oral cavity and pharynx (C00–C14), esophagus (C15), colorectal (C18–C20), liver (C22), thyroid (C73), gall bladder (C23–C24), pancreas (C25), and larynx (C32) in both sexes; and breast (C50) in females (Table 1). We obtained age and sex-specific estimates of the number of new cancer cases reported by the individual registries from their cancer registry databases for the time period under review (Table 1).

**Table 1:** Population attributable fraction (PAF) and estimated numbers of cancers attributable to alcohol consumption in Nigeria from 2012 to 2014.

Cancer site	ICD-O3 code	No. of cancer cases	% of alcohol associated cancers	ASR	PAF%	Cancer cases attributable to alcohol	ASR
**Male**							
Oral cavity and pharynx	C00–C14	79	4.4	4.2	44.7	36	1.5
Esophagus	C15	12	0.7	0.6	51.8	6	0.3
Colorectal	C18–C20	107	5.9	5.1	15.0	16	1.0
Liver	C22	93	5.1	4.0	13.0	12	0.5
Gall bladder	C23–C24	3	0.2	0.2	25.3	1	0.1
Pancreas	C25	19	1.1	1.0	5.4	1	0.1
Larynx	C32	26	1.4	1.1	28.4	7	0.3
Total cancers in males (%)		339 (20.8)	18.8	16.2		79 (4.9)	4.3
**Female**							
Oral cavity and pharynx	C00–C14	57	3.1	5.0	17.2	10	1.0
Esophagus	C15	7	0.4	0.5	28.3	2	0.1
Colorectal	C18–C20	97	5.4	7.0	2.3	2	0.1
Liver	C22	52	2.9	2.7	12.8	7	0.4
Gall bladder	C23–C24	4	0.2	0.3	8.2	0	0.0
Pancreas	C25	27	1.5	2.0	1.4	0	0.0
Larynx	C32	8	0.4	0.6	9.7	1	0.1
Breast	C50	1,217	67.3	60.1	7.3	85	4.2
Total cancers in females (%)		1,469 (54.2)	81.2	78.2		107 (3.9)	5.9
Total cancers in both sexes (%)		1,808 (41.7)	41.7	77.3		186 (4.3)	7.2

We used the population attributable fraction (PAF) computed for each cancer site by age and sex category from the formula (15):

$$\sum \frac{(P*\left(\text{RR}-1\right)}{\left(P*\left(\text{RR}-1\right)+1\right)}$$

where RR represents the relative risk of alcohol consumption, and *P* is the proportion of people who consume alcohol in the population. This method has been used in similar studies to estimate the number of cancers attributable to alcohol consumption (9–13).

For this study, we used the PAF for alcohol consumption associated with cancers derived from prevalence and relative risk estimates worldwide (Table 2) (4). We did not use PAF from Nigeria because there were no relative risks for different categories of alcohol consumption (light drinkers ≤ 1 drink/day, moderate 2–3 drinks/day, and heavy drinkers > 4 drinks/day) in our population. We calculated the numbers of cancer cases attributable to alcohol consumption in each sex by multiplying the PAF of each cancer site with the overall numbers of cancer cases reported per cancer site according to the formula (15):

ACalc=∑(PAF∗N)

where *N* represents the number of alcohol associated cancers per cancer site reported by the PBCRs. We carried out sensitivity analyses for cancers attributable to alcohol, using GLOBOCAN 2012 database for Nigeria (Table 2). The GLOBOCAN is a data source from IARC on estimated cancer incidence, mortality, and prevalence for 184 countries of the world including Nigeria in 2012. GLOBOCAN 2012 estimated cancer incidence in Nigeria by using regional incidence rates to estimate the weighted average of local incidence rates from three cancer registries in Nigeria (Abuja, Ibadan, and Enugu) for the time period 2006–2011 (16).

**Table 2:** Sensitivity analyses of cancers attributable to alcohol consumption in Nigeria from GLOBOCAN 2012 database.

Cancer site	ICD-O3 code	No. of cancer cases	ASR	PAF%	Cancer cases attributable to alcohol	ASR
**Male**						
Oral cavity and pharynx	C00–C14	1,441	10.4	44.7	648	4.7
Esophagus	C15	145	9.0	51.8	75	4.7
Colorectal	C18–C20	2,164	20.6	15.0	325	3.1
Liver	C22	7,875	15.3	13.0	1,024	2.0
Gall bladder	C23–C24	90	2.1	25.3	23	0.5
Pancreas	C25	935	4.9	5.4	47	0.2
Larynx	C32	732	3.9	28.4	205	1.1
Total cancers in males (%)		13,382 (35.8)	66.2		2,347 (6.3)	16.3
**Female**						
Oral cavity and pharynx	C00–C14	1081	4.0	36.7	400	1.5
Esophagus	C15	141	3.1	44.7	63	1.4
Colorectal	C18–C20	2,008	14.3	9.2	181	1.3
Liver	C22	4,172	5.3	12.9	542	0.7
Gall bladder	C23–C24	88	2.3	15.6	14	0.4
Pancreas	C25	738	3.6	3.5	30	0.1
Larynx	C32	59	0.5	26.1	15	0.1
Breast	C50	27,304	43.1	7.3	1,911	3.0
Total cancers in females (%)		35,591 (55.0)	76.2		3,156 (4.9)	8.5
Total cancers in both sexes (%)		48,973 (48.0)	92.8		5,503 (5.4)	13.9

### Ethics

The Nigerian National Health Research Ethics Committee determined that cancer registration activities are exempt from review and informed consent from participants is not required because researchers do not access identifiable patient information (17). We used de-identified data for our study and did not have access to participants’ personal health information.

## Results

The two PBCRs reported 4,336 new cancer cases from 2012 to 2014 (ASR 113.9 per 100,000) of which 1,627 (37.5%; ASR 82.0 per 100,000) occurred in males while 2,709 (62.5%; ASR 145.9 per 100,000) occurred in females. Of these, 1,808 (41.7%) were alcohol associated with an ASR of 77.3 per 100,000. Some 81.3% (1,469 of 1,808; ASR 78.2 per 100,000) of the alcohol associated cancers occurred in females while 18.8% (339 of 1,808; ASR 16.2 per 100,000) occurred in males (Table 1).

Overall, only 4.3% (186 of 4,336; ASR 7.2 per 100,000) of the cancers were attributable to alcohol consumption. Most of the cancers attributable to alcohol (2.5%; 107/4336; ASR 5.9 per 100,000) occurred in women and (1.8%; 79/4336; ASR 4.3 per 100,000) occurred in men.

### Oral Cavity and Pharyngeal Cancer

**Figure 1:**
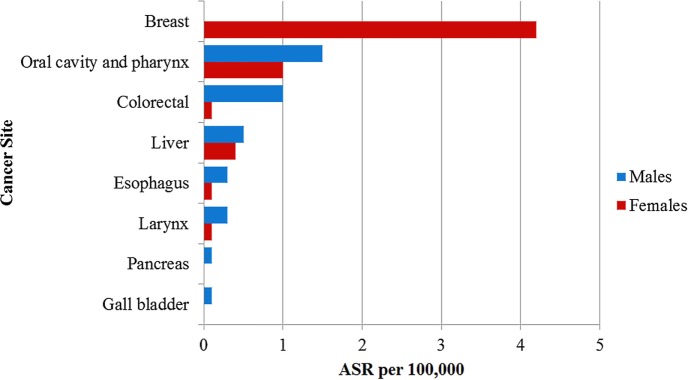
Age standardized incidence rate per 100,000 of cancers attributable to alcohol consumption in Nigeria reported by the population-based cancer registries.

The PBCRs reported 136 oral cavity and pharyngeal cancers from 2012 to 2014 (ASR 4.6 per 100,000). Of these, 79 (58.1%; ASR 4.2 per 100,000) cases occurred in males and 57 (41.9%; 5.0 per 100,000) cases occurred in females. Using the PAF estimates of 44.7% in males and 17.2% in females (4), we computed that 33.8% (46/136; ASR of 1.5 per 100,000) of these cancers were attributable to alcohol consumption (Table 1). The ASR for cancers of the oral cavity and pharynx attributable to alcohol consumption were 1.5 per 100,000 in males and 1.0 per 100,000 in females (Figure 1).

### Esophageal Cancer

There were 19 esophageal cancer cases in both sexes from 2012 to 2014 (ASR 0.6 per 100,000), of which 12 (63.2%; ASR 0.6 per 100,000) occurred in males while 7 (36.8%; ASR 0.5 per 100,000) occurred in females. We calculated the alcohol attributable fraction for cancer of the esophagus using PAF estimates of 51.8% in males and 28.3% in females and found that 42.1% (8/19; ASR 0.2 per 100,000) of the esophageal cancers in both sexes were attributable to alcohol consumption (Table 1) (4). The ASR for esophageal cancers attributable to alcohol was 0.3 per 100,000 in males and 0.1 per 100,000 in females (Figure 1).

### Colorectal Cancer

A total of 204 colorectal cancer cases were reported in both sexes from 2012 to 2014 by ABCR and ECR (ASR 6.1 per 100,000). Colorectal cancer was the most common alcohol associated cancer in males, representing 5.9% (107/1,808; ASR 5.1 per 100,000) of male cancers and the second most common alcohol associated cancer in females constituting 5.4% (97/1,808; ASR 7.0 per 100,000) female cancers. Using the PAF estimates of 15.0% in males and 2.3% in females (4), 8.8% (18/204; ASR 0.6 per 100,000) of colorectal cancer cases were attributable to alcohol consumption (Table 1). The ASR for colorectal cancers attributable to alcohol was 1.0 per 100,000 in males and 0.1 per 100,000 in females (Figure 1).

### Liver Cancer

The registries reported 145 liver cancer cases (ASR 3.4 per 100,000) with 93 (64.1%; ASR 4.0 per 100,000) cases in males and 52 (35.9%; ASR 2.7 per 100,000) cases in females. Using a PAF estimates of 13.0% in males and 12.8% in females (4), we computed that 19 of the 145 liver cancer cases (13.1%; ASR 0.5 per 100,000) were attributable to alcohol consumption (Table 1). The ASR for liver cancers attributable to alcohol consumption was 0.5 per 100,000 in males and 0.4 per 100,000 in females (Figure 1).

### Gall Bladder Cancer

We found seven gall bladder cancer cases from 2012 to 2014 (ASR 0.3 per 100,000) in the PBCRs data. Of these, three cases (42.9%; ASR 0.2 per 100,000) occurred in males and four cases (57.1%; ASR 0.3 per 100,000) occurred in females. With a PAF estimates of 25.3% in males and 8.2% in females (4), we computed that one of the seven gall bladder cancer cases in males (14.3%; ASR 0.1 per 100,000) was attributable to alcohol consumption (Table 1). There was no gall bladder cancer case attributable to alcohol consumption in females.

### Pancreatic Cancer

The PBCRs reported 46 cases of pancreatic cancers in the study period (ASR 1.5 per 100,000) of which 19 (41.3%; 1.0 per 100,000) cases occurred in males while 27 (58.7%; 2.0 per 100,000) cases occurred in females. Using a PAF estimates of 5.4% in males and 1.4% in females (4), we estimated that 1 of the 46 pancreatic cancer cases in males (2.2%; ASR 0.1 per 100,000) was attributable to alcohol consumption (Table 1). There was no pancreatic cancer case attributable to alcohol consumption in females.

### Laryngeal Cancer

Some 34 laryngeal cancer cases were reported by ABCR and ECR within the study period (ASR 0.9 per 100,000). Of these, 26 (76.5%; ASR 1.1 per 100,000) cases occurred in males and 8 (23.5%; ASR 0.6 per 100,000) cases occurred in females. In our study, we calculated the alcohol attributable fraction for cancer of the larynx using a PAF estimates of 28.4% in males and 9.7% in females (4). We computed that 23.5% (8/34; ASR 0.2 per 100,000) of these cancers were attributable to alcohol consumption (Table 1). The ASR for laryngeal cancers attributable to alcohol was 0.3 per 100,000 in males and 0.1 per 100,000 in females (Figure 1).

### Breast Cancer

Breast cancer was the commonest alcohol associated cancer reported during the study period. A total of 1,217 cases were reported in females with ASR 60.1 per 100,000, representing 28.1% (1,217/4,336) of all new cancers reported by the registries from 2012 to 2014. Using a PAF estimates of 7.3% (4), we estimated that 2.0% (85/4,336; ASR 4.2 per 100,000) of breast cancer cases reported within the study period were attributable to alcohol consumption (Table 1). There was no breast cancer case attributable to alcohol consumption in males.

### Thyroid Cancer

The PBCRs reported 44 cases of thyroid cancers in both sexes from 2012 to 2014. Of these, 9 (20.5%) cases occurred in males, while 35 (79.5%) cases occurred in females. The combined ASR for thyroid cancers was 1.2 per 100,000; ASR for males was 0.4 per 100,000 and 2.0 per 100,000 for females. To determine how much more cancers of the thyroid there may have been if the people stop taking alcohol, we computed the association between mild to moderate alcohol intake and reduced risk of thyroid cancer using a relative risk of 0.74 from meta-analyses studies (18). We estimated that 60 cases of thyroid cancers with ASR 1.6 per 100,000 in both sexes (ASR 0.5 per 100,000 for males and 2.6 per 100,000 for females), would have occurred if alcohol was not consumed.

### Sensitivity Analyses

In GLOBOCAN 2012, it was estimated that 102,079 cancer cases occurred in Nigeria [37,370 (36.6%) in males and 64,709 (63.3%) in females]. Of these, a total of 48,973 (48.0%; ASR 92.8 per 100,000) cancer cases, 27.3% (13,382/48,973) in males and 72.7% (35,591/48,973) in females, were associated with alcohol consumption. Of all incident cancers reported by GLOBOCAN, 5.4% (5,503/102,079; ASR 13.9 per 100,000) were attributable to alcohol consumption [2,347 (6.3%) in males, 3,156 (4.9%) in females] (Table 2).

In both data sources, the most common cancer attributable to alcohol consumption was breast cancer which accounted for approximately 2.0% of all cancer cases (85 of 4,336 in data from Nigeria PBCRs and 1,911 of 102,079 in data from GLOBOCAN 2012).

## Discussion

This is the first study to our knowledge, to report on the burden of cancers attributable to alcohol consumption in Africa. In this study, we estimated that 4.3% of all cancer cases in both sexes, 4.9% in men and 3.9% in women were attributable to alcohol consumption. The cancers with incidence most impacted by alcohol consumption were cancers of the oral cavity and pharynx in men (ASR 1.5 per 100,000) and cancer of the breast in women (ASR 4.2 per 100,000). It is noteworthy that among women in our study, 80% (85/107) of all cancers attributable to alcohol consumption occurred in the breast.

The consumption of alcohol in Nigeria varies significantly by gender and age. Other factors such as religious and cultural norms influence the consumption of alcohol (19). However, with socio-economic development, the volume and types of alcohol being consumed in the country has been increasing (19). According to the WHO, Nigeria ranks 27th worldwide in volume of alcohol in liters, consumed per capita per year, and it is the fourth leading country in alcohol consumption in Africa (2).

Alcohol consumption has been linked to the development of cancers of the oral cavity, pharynx, larynx, esophagus, liver, colon, rectum, and breast (1, 13). A recent review by the International Head and Neck Cancer Epidemiology consortium reports a multiplicative effect on the risk of oral and pharyngeal cancers (OPC) in people who smoke and drink alcohol (20). Other risk factors for OPC include oral sex and human papilloma virus infection (21).

Esophageal cancer is one of the most common cancers in East and Southern Africa (22), but it is less common in West Africa (23) and remains rare in Nigeria (24). Studies done to find out the reason for this uneven distribution across sub-Saharan Africa (SSA) regions have been inconclusive (25, 26). It has been suggested that these regional differences may be due to differences in risk factor profiles across SSA (27). Some studies have shown that unlike the Western African population, more people in the Eastern Africa consume locally brewed spirits or beer, and the peculiar process of brewing the drinks could predispose consumers to esophageal cancer (25, 28). The synergistic effect of heavy alcohol consumption and smoking habits which is a risk factor of esophageal cancer could be a potential factor for the disparities in incidence of esophageal cancer in the Eastern and Western Africa (25). Other studies have suggested that gene environmental interactions could contribute to increased risk of esophageal cancer in different regions (29, 30).

In Nigeria, colorectal cancer is one of the most common cancers in both sexes (31). It disproportionately affects men in Nigeria (31) and worldwide (32). Colorectal cancer was the most common alcohol associated cancer in males and the second most common in females in this study and the ASRs are similar to rates reported in Southern Africa but higher than in East and Central Africa (33). The WHO Status Report on Alcohol reveals that there has been a significant steep rise in alcohol consumption in Nigeria over the past 15 years, with increase in wine consumption, slight increase in beer consumption, while spirit consumption has remained the same. These could have contributed to the rising incidence of colorectal cancer in this population (2, 34). Other risk factors for colorectal cancer include dietary and lifestyle changes such as consumption of processed meat, smoking, and genetic predisposition (31, 35). Exposure of colon mucosa to acetaldehyde from microbial metabolism of ethanol in alcoholic drinks has been postulated as a mechanism for increasing the risk of developing colorectal cancer (36).

Liver cancer is the third commonest cancer in Nigerian men after prostate and colorectal cancers (31), and is a common cancer in other parts of West Africa (37, 38). It contributed to approximately one-third of all alcohol associated cancers in men in this study. Its risk factors include long-term alcohol use, cigarette smoking, dietary aflatoxins, and Hepatitis B and C virus infections (39, 40). It has been estimated that 47% of all liver cancers in SSA are attributable to Hepatitis infections while a significant proportion of the remainder are due to alcohol consumption (41). The predominance of liver cancers in men is thought to be due to the higher rate of risk factors including viral hepatitis, cigarette smoking, and alcohol consumption in men than in women (37).

Breast cancer is the most common cancer among Nigerian women and the second most common cancer in SSA (7). Risk factors, including age, sex, early age at menarche, parity, breast feeding, use of combined oral contraceptive pills, family history, diet, height, truncal obesity, and postmenopausal weight gain are responsible for most of the risk associated with breast cancer (42, 43). However, because of its high incidence, breast cancer was the most common alcohol associated cancer in this study. Nevertheless, the proportion of breast cancers attributable to alcohol consumption was low compared with the total number of breast cancer cases reported by the registries. Traditionally, alcohol consumption is less common among women in SSA however, with the increasing influence of western culture and lifestyles, more women are now getting exposed to alcohol, which may increase the risk of breast cancer in this population in the future (44, 45).

In contrast to the increased risk of the several cancers with alcohol consumption, the risk of thyroid cancer decreases with light and moderate alcohol intake (8, 18, 46). Alcohol intake may have a protective effect on developing thyroid cancer by decreased levels of thyroid-stimulating hormone, the growth factor associated with thyroid cancer (47). Another potential biological mechanism is that alcohol may have a direct toxic effect on thyroid cells and reduce thyroid volume, which might lead to a decreased risk of thyroid cancer (48).

In general, while there are many publications on the association between alcohol intake and cancers, most of the studies have not focused carefully on the relationship with the amount consumed and the duration of consumption. Thus, although associations between several cancers and high alcohol intake is known, these associations become less clear for low to moderate intake of alcohol (49). Few countries in Africa have policies regulating alcohol consumption (19). Public health interventions on the risks or benefits of alcohol intake need to be based on high quality data about its impact on diseases.

The limitations of our study include our inability to rule out incompleteness of cancer registration and underreporting of some alcohol associated cancers such as cancers of the liver, colon, and rectum due to lack of awareness, lack of presentation in hospitals, and inability to biopsy certain cancers due to limited facilities for accurate diagnosis. However, our sensitivity analyses show that our findings are similar to estimates for alcohol associated cancers in Nigeria as reported in the GLOBOCAN 2012 database of the IARC. Our results may be conservative estimates of the total burden of alcohol associated cancers in Nigeria given that we have restricted our analysis to cancers with an established association with alcohol drinking and excluded other cancers for which there is a less clear relationship such as lung and kidney cancers.

## Conclusion

We present the first study conducted on the burden of alcohol associated cancers and the proportion attributable to alcohol consumption in the Nigerian population. This study sets the foundation for future studies in this field to document secular trends and enable appropriate public health interventions.

## Author Contributions

MO and EJ-A contributed to data collection, data analyses, and drafting the manuscript. All authors contributed to the writing of this manuscript. ED and SA contributed to data interpretation and revising it critically for intellectual content. EO, FI, TO, EE, and RH contributed to data collection and data quality. CA conceived the idea for the study, obtained funding, provided critical revisions, and guided all aspects of the paper. All authors read and approved the final manuscript.

## Conflict of Interest Statement

The authors declare that the research was conducted in the absence of any commercial or financial relationships that could be construed as a potential conflict of interest.
